# Protective effect of canagliflozin on post-resuscitation myocardial function in a rat model of cardiac arrest

**DOI:** 10.1186/s40635-023-00562-y

**Published:** 2023-11-15

**Authors:** Tianfeng Hua, Yuqian Chu, Minjie Wang, Yijun Zhang, Wei Shi, Qihui Huang, Liangliang Zhang, Min Yang

**Affiliations:** The Second Department of Critical Care Medicine and The Laboratory of Cardiopulmonary Resuscitation and Critical Care, The Second Affiliated Hospital of Anhui Medical University, 678 Furong Road, Hefei, 230601 Anhui Province China

**Keywords:** Canagliflozin, Cardiac arrest, Post-resuscitation myocardial function, Myocardial ischaemia/reperfusion injury

## Abstract

**Background:**

Currently, most patients with cardiac arrest (CA) show reversible myocardial dysfunction, hemodynamic instability, systemic inflammation and other pathophysiological state in early stage of resuscitation, some patients may eventually progress to multiple organ failure. There is evidence that heart failure is the terminal stage in the development of various cardiovascular diseases. Although the cardio-protective effect of canagliflozin (CANA) has been confirmed in large clinical studies and recommended in domestic and international heart failure-related guidelines, the effectiveness of CANA after resuscitation remains unclear. In this study, we constructed a modified CA/CPR rat model to investigate whether CANA administered on post-resuscitation improves myocardial function.

**Methods:**

Twenty-fourth healthy male Sprague–Dawley rats were randomized into four groups: (1) Sham + placebo group, (2) Sham + CANA group, (3) CPR + placebo group, and (4) CPR + CANA group. Ventricular fibrillation was induced by transcutaneous electrical stimulation on epicardium. After 6 min untreated ventricular fibrillation, chest compressions was initiated. The rats were received an injection of placebo or canagliflozin (3 ug/kg) randomly 15 min after restore of spontaneous circulation (ROSC). Electrocardiogram (ECG) and blood pressure were continuously detected in each group throughout the experiment. The rats were killed 6 h after ROSC to collected the arterial serum and myocardial tissue. Myocardial injury was estimated with concentrations of inflammatory factors, oxidative stress indexes and, apoptosis index, myocardial injury markers, echocardiography and myocardial pathological slices.

**Results:**

After resuscitation, mean arterial pressure (MAP) were significantly increased after cardiopulmonary resuscitation in CANA group rats when compared with placebo group. Heart rate, body lactate returned and left ventricular ejection fraction (LVEF) to normal levels in a shorter time and the myocardial injury was obviously attenuated in CPR + CANA group. Inflammatory factors (IL-6, TNF-α) and oxidative stress indexes (MAD, SOD, CAT) were dramatically decreased with the administration of CANA. The expression of apoptosis index (BAX, caspase-3) were higher in CPR + placebo group and the expression of anti-apoptosis index (Bcl-2) was lower (*P* < 0.05).

**Conclusions:**

The administration of CANA effectively reduces myocardial ischaemia/reperfusion (*I*/*R*) injury after cardiac arrest and cardiopulmonary resuscitation (CPR), and the underlying mechanism may be related to anti-inflammation, oxidative stress and apoptosis.

Cardiac arrest (CA) is a global health issue, accounting for approximately 1/5 of adult deaths in the United States each year [1]. Despite rapid advances in medical technology, up to 50% of resuscitated patients still die before discharge from the hospital [2]. More than 2/3 of patients with ventricular fibrillation-related cardiac arrest have hemodynamic disorders, considered as post-resuscitation myocardial dysfunction (PRMD), the leading cause of early death after recovery of spontaneous circulation (ROSC) [3–5].

PRMD occurs in approximately 68% of post-resuscitation patients which pathophysiological mechanism is complex [6, 7]. During cardiac arrest, the body's energy metabolism and oxygen transport are in a state of suspended animation. However, chest compressions provide only limited perfusion pressure, which cannot counteract the ischaemia caused by microthrombus and vasospasm [8]. The heart undergoes cardiac arrest and CPR leads to myocardial I/R injury. In pathological conditions, cardiomyocyte death or injury releases endogenous signals known as danger-associated molecular patterns (DAMPs) [9, 10], which in turn release a variety of pro-inflammatory chemokines and cytokines, initiate and regulate innate immune responses. Recently, the adaptive immune response has been highlighted as another significant factor in MIRI [11, 12]. However, uncontrolled or excessive inflammatory factors can aggravate tissue injury. In addition, a large number of oxygen-free radicals disrupt the dynamic balance between oxidative and antioxidant systems. Excessive reactive oxygen species (ROS) cause the damage to different membrane structural components and harm cellular physiological functions. For example, ROS lead to electrophysiological impairment and contractile dysfunction of cardiomyocytes by affecting the Na + /Ca2 + exchanger (NCX), the L-type calcium channel and the sarcoplasmic reticulum Ca2 + -ATPase [13]. On the other hand, inflammatory factors increase vascular permeability and induce neutrophil adhesion, which increases the expression of pro-apoptotic factors. Inflammation can also activate the death receptor pathway and induce cardiomyocyte apoptosis [14, 15].

Several large clinical trials (CANVAS, CREDENCE) have shown that CANA can significantly reduce cardiovascular events [16, 17]. International guidelines recommend that CANA are considered as first-line therapy in patients with type 2 diabetes mellitus (T2DM) at high cardiovascular risk [18, 19]. A post-hoc analysis of the CANVAS trial showed that CANA reduced plasma IL-6 levels in people at high cardiovascular risk [20], and Heerspink et al. also found the same anti-inflammatory properties of CANA [21]. Peyton et al. demonstrated that CANA inhibits the inflammatory response of human endothelial cells by regulating HO-1 expression. In addition to its anti-inflammatory properties, CANA also reduces mitochondrial ROS production, thereby preserving the functional integrity of endothelial cells [22]. Several studies have shown that SGLT2 inhibitors significantly reduce oxidative stress markers in myocardial tissue with and without diabetes [23, 24]. In addition, more and more studies have found the anti-apoptotic effect of CANA in many diseases. CANA can inhibit carfilzomib (CFZ)-induced endothelial cell apoptosis via the AMPK pathway for the adjuvant treatment of relapsed/refractory multiple myeloma [25]. It can also inhibit apoptosis by down-regulating CX3CL1 expression in the heart and kidney to treat cardiorenal syndrome [26]. There is increasing evidence that CANA has anti-inflammatory, anti-apoptotic, and antioxidant properties. Therefore, we hypothesize that CANA may improve post-resuscitation cardiac dysfunction in CA/CPR patients.

Studies on the effects of CANA in patients with cardiac arrest are still at an early stage, and the specific mechanisms are still unclear. We hypothesize that CANA is effective in attenuating myocardial I/R injury after cardiac arrest and resuscitation, and that the underlying mechanisms are related to anti-inflammation, oxidative stress and apoptosis.

## Methods

### Design

This was a randomized controlled study in rats to investigate the effect of intravenous CANA on cardiac function after cardiopulmonary resuscitation. The Animal Experiment Ethics Committee of Anhui Medical University approved this study. All experimental animals were treated according to the Guide for the Care and Use of Laboratory Animals (NIH Publication No. 85-23, revised 1996).

### Anaesthesia and animal preparation

Twenty-fourth healthy male Sprague–Dawley rats, 8–10 months, weighing 400–450 g, were provided by the Laboratory Animal Center of Anhui Medical University. The rats were housed in a temperature-controlled room and maintained on a 12-h diurnal rhythm with adequate water and food. After inhalation of carbon dioxide (CO_2_) for approximately 30 s to induce anesthesia, the animals were injected intraperitoneally with pentobarbital at an initial dose of 45 mg/kg. A maintenance dose of 10 mg/kg was administrated as needed. The trachea of the animals was orally intubated with a 14G cannula mounted on a blunt needle (Abbocath-T; Abbott Hospital Products Division, United States) with a 145°angled tip. The rats were mechanically ventilated with a multichannel small-animal ventilator (KW-100-2, NJKEWBIO, China) was used for mechanical ventilation, set at a tidal volume of 0.6 mL/100 g body weight, a rate of 100 breaths/min, and an inspired oxygen fraction of 0.21. Two PE-50 catheters (PE50, Smith Medical, United Kingdom) were prepared for cannulation of the left femoral artery and vein, the left femoral artery catheter to the physiological monitor for the measurements of vital signs, and the collection of blood samples before sacrificing rats. The venous catheter is used to establish an intravenous infusion route for the administration of CANA or placebo. All catheters were primed with 2.5U/ml heparinized saline. ECG recording with lead II and heart rate monitoring throughout the experiment. During the experiment, the rectal temperature was maintained at 37 ± 0.5℃ with a heating lamp.

### Experimental protocol

Twenty-fourth male rats were randomly divided into four groups: (1) CPR + CANA group (*n* = 6): canagliflozin was administered by intravenous injection (dose: 3 µg/kg) after 15 min of recovery of spontaneous circulation (ROSC). (2) CPR + placebo group (*n* = 6): equal volumes of placebo were administered by intravenous injection at the same timepoint. (3) Sham + CANA group (*n* = 6): the equal dose of canagliflozin was administered. (4) Sham + placebo group (*n* = 6): equal volumes of placebo were administered.

**Fig. 1:**
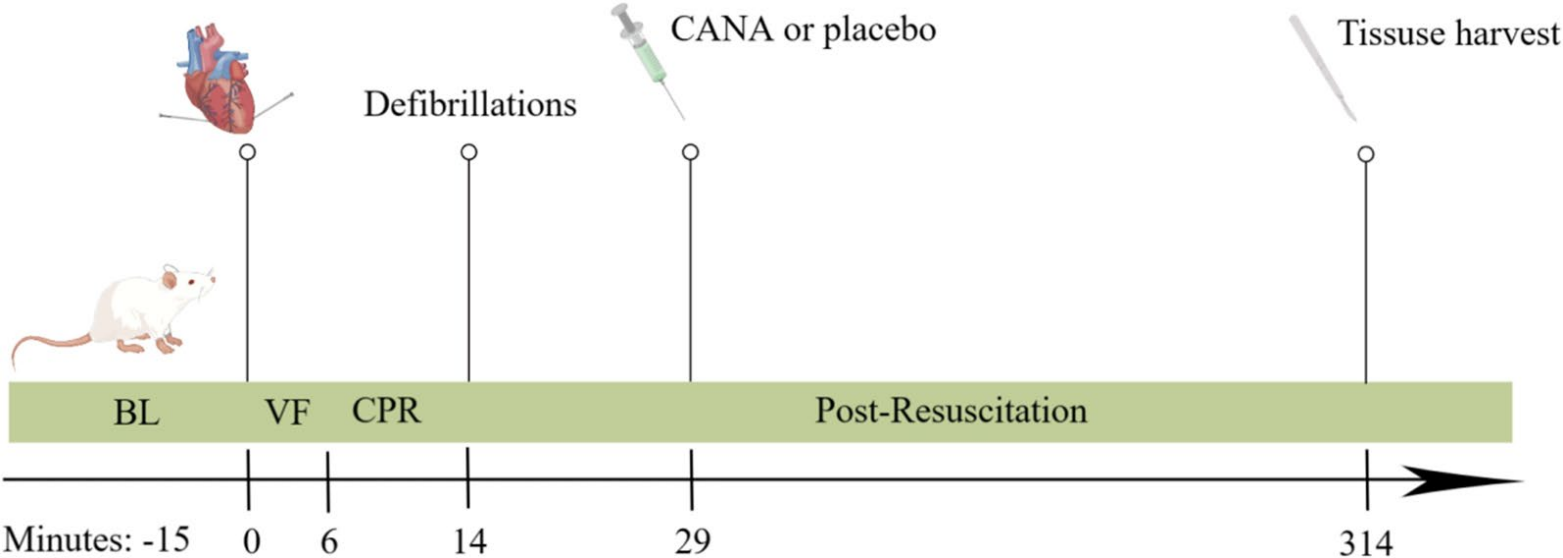
Experimental procedure. 15 means 15 min before ventricular fibrillation was induced; *BL* baseline, *VF* ventricular fibrillation, *CPR* cardiopulmonary resuscitation, *CANA* canagliflozin

**Fig. 2:**
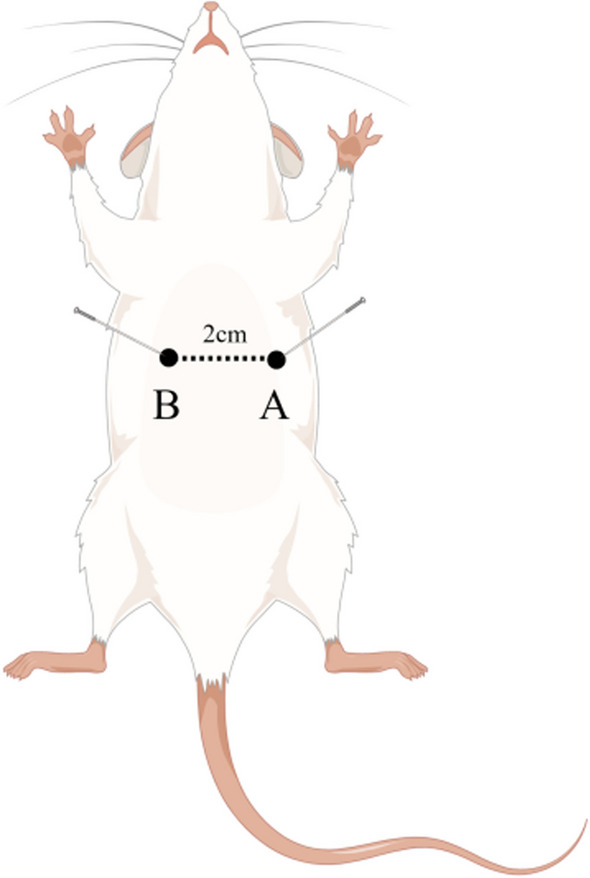
Sketch of point A/B

**Fig. 3:**
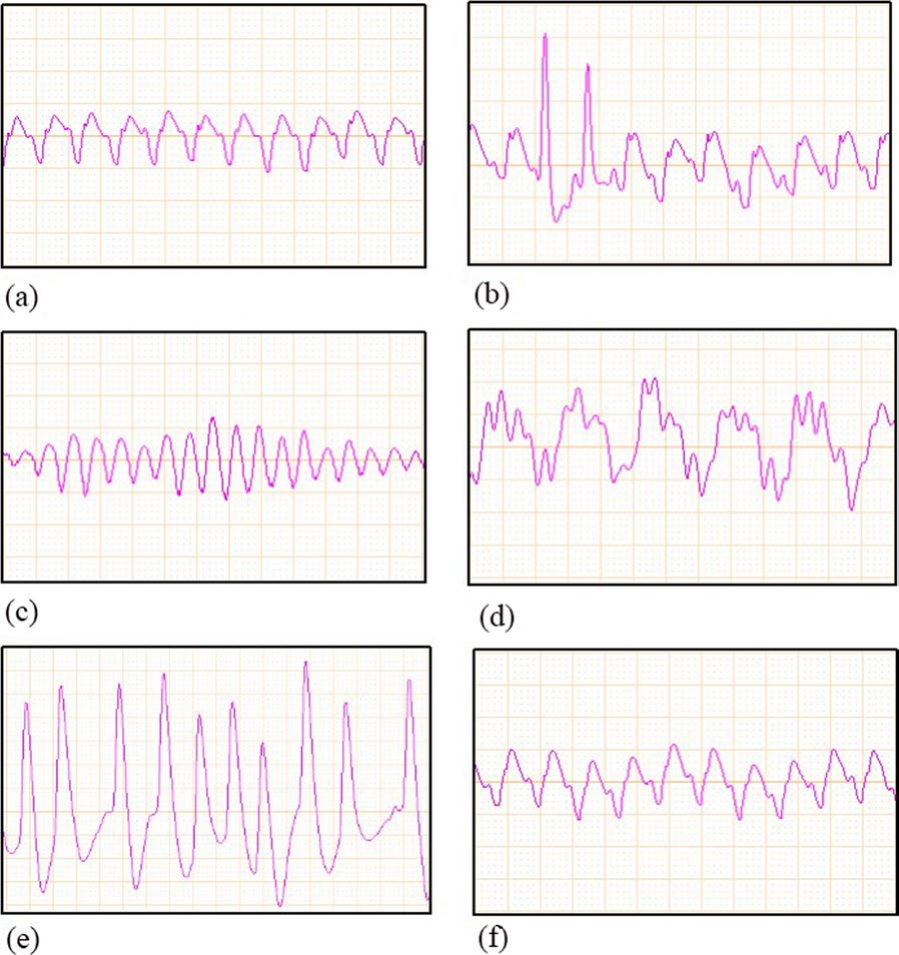
ECG during experiment. **a** Normal ECG, **b** ECG of needle insertion, **c** ECG during induction of VF, **d** ECG during CPR, **e** ECG of post-resuscitation arrhythmias, **f** normal ECG of post-resuscitation

The experimental procedure is shown in Fig. 1. Baseline vital signs (heart rate and blood pressure) and arterial blood gases (RAPID Point500, SIEMENS, Germany) were recorded 15 min before induction of ventricular fibrillation (VF). VF was induced by acupuncture needles inserted into epicardial electrical stimulation. Point A is positioned at the most vital point of the cardiac apex beat of a rat; point B is set at approximately 2.0 cm horizontally to the right of point A. Points A and B are the needle entry points for epicardial electrical stimulation (Fig. 2). The needle insertion angle of point A is approximately 60°, while the needle insertion angle of point B is approximately 75°, and the insertion depth is approximately 1.5–2.0 cm. When the needle tip reaches the epicardium, the needle tail has a rapid swinging action consistent with the beating frequency of the rat's heart, and the rat's ECG would show a transient arrhythmia. There may also be a transient drop in blood pressure. Small animal cardiopulmonary resuscitation instruments (KW-XF, NJKEWBIO, China) were used for the instrument construction of CA/CPR model. The direct current was progressively increased from an initial 0.5 mA to a maximum of 1.5 mA. In addition, the stimulation was maintained for 3 min to prevent spontaneous cardioversion. Mechanical ventilation was stopped throughout the VF process. Following 6 min of untreated VF, chest compressions (compression rate of 200 times/min, compression depth of 1.0–1.3 cm) and mechanical ventilation (inspired oxygen fraction of 1.0) were initiated and maintained for 8 min. Defibrillation was performed with up to 4 J countershocks. Successful defibrillation is indicated by ROSC, defined as MAP ≥ 50 mmHg maintained for 5 min. If defibrillation fails, continue compressions for 30 s and then defibrillation again. Failure to achieve ROSC after more than 3 cycles is considered as a failure. After ROSC, an inspired oxygen fraction of 1.0 was maintained for 1 h, adjusted to 0.5 for another hour, and then to 0.21. Detailed ECG changes during the experiment are shown in Fig. 3.

Echocardiograms were obtained at baseline and at post-resuscitation 2 h, 4 h, and 6 h. At the end of 6 h after ROSC, animals were sacrificed with an intravenous administration of 150 mg/kg pentobarbital. Myocardial tissue and serum samples were collected and stored in a – 80 ℃ refrigerator for further analysis. In addition, each rat was necropsied to check for any severe injury due to improper handling.

## Measurements

### Basic vital signs

ECG, heart rate (HR), mean aortic pressure (MAP), body temperature were continuously recorded on a personal computer-based biological signal acquisition and processing system (Medlab-U/4C501H, NJKEWBIO, China). Left ventricular ejection fraction (LVEF) was measured by echocardiography (6LAB, VINNO, China) at baseline and at post-resuscitation 2 h, 4 h, and 6 h.

### Myocardial histopathological damage

Myocardial tissue was fixed in 10% neutral formalin for 24 h. Paraffin was used for routine embedding, and the embedded specimens were sectioned at 4 μm thickness. After sectioning, the slices were baked in an oven for 12 min and then placed in an automatic hematoxylin and eosin (H&E) staining machine for HE staining. Myocardial pathology images were captured using an upright biological microscope (Axio Scope A1, Carl Zeiss, Germany) and imaging software at 400* magnification. The degree of myocardial edema, inflammatory cell infiltration, and myocardial fiber arrangement were observed to determine myocardial injury. Myocardial histopathological damage was graded independently by three independent observers.

### Assessment of related protein expression

After ROSC 6 h, arterial blood and extracted supernatant of animals were collected for determining relevant inflammatory markers (IL-6, TNF-α), oxidative stress markers (SOD, MDA, CAT) and myocardial injury markers (NT-Pro BNP, cTnI). IL-6, TNF-α, NT-Pro BNP and cTnI were measured with commercial ELISA kits according to the manufacturer’s instructions (E-EL-R0015c/E-EL-R2856c/E-EL-R3023/E-EL-R1253c, Elabscience Biotechnology Co., Ltd, China), respectively. SOD, MDA and CAT were measured with commercial ELISA kits according to the manufacturer’s instructions (JYM0990Ra/JYM0266Ra/JYM1143Ra, Wuhan ColorfulGene Biological Technology Co., LTD, China), respectively.

An appropriate amount of myocardial tissue samples was weighed, ground. In addition, total proteins were extracted in grinding reagent with 500ul RIPA (P0013B, Beyotime Biotechnology, China) + 5 μl PMSF (ST505, Beyotime Biotechnology, China) per 100 mg of tissue. The concentration of extracted protein was determined by BCA protein assay kit. Then, 5 × loading buffer (P0015L, Beyotime Biotechnology, China) was added and boiled for 10 min. The protein homogenate was separated by sodium dodecyl sulfate (SDS)–polyacrylamide gel electrophoresis and then transferred to the PVDF membrane (IPVH00010, Merck Millipore, Germany) using a membrane transfer device. The membrane was blocked with 5% skim milk powder for 2 h, washed, and incubated with primary antibodies overnight at 4℃: BAX (1:1000; ab32503, Abcam, United Kingdom), Bcl-2 (1:500; GTX100064, Gene Tex, United States), Caspase-3 (1:2000; ab184787, Abcam, United Kingdom), Nrf2 (1:500; AF0639, Affinity Biosciences, United States), HO-1 (1:500; AF5393, Affinity Biosciences, United States), GAPDH (1:5000; AF0911; Affinity Biosciences, United States). After washing, they were incubated with secondary antibody (1:1 0000; E-AB-1034; Elabscience Biotechnology Co., Ltd, China) for 1 h at room temperature. The gray value of the target band was analyzed using Image J, and the expressions level of the target proteins were expressed as the ratio of the gray value of the target band to the gray value of the GAPDH band.

### Statistics

SPSS version 19.0 statistical software was used to analyze all data. Measurement data conforming to normal distribution are expressed as mean ± SD. One-way ANOVA was used for intergroup comparisons, and the SNK-q test was used for pairwise comparisons. The Chi-squared test was used to compare enumeration data between groups. A value of *P* < 0.05 was considered statistically significant.

## Results

A total of 24 rats were used and analyzed. There was no difference in baseline characteristics between the four groups of rats (Table 1). All rats that received epicardial fibrillation were successfully resuscitated and survived.

**Table 1:** Baseline characteristics

Group	Sham + placebo	Sham + CANA	CPR + placebo	CPR + CANA
Body weight (g)	468.00 ± 17.7	475.83 ± 21.3	461.50 ± 19.7	467.25 ± 19.3
Heart rate (bpm)	411.17 ± 28.4	417.83 ± 25.3	419.33 ± 30.2	426.5 ± 34.5
MAP (mmHg)	114.00 ± 7.4	121.00 ± 8.6	119.00 ± 11.4	118.83 ± 15.0
Lactate (mmol/L)	0.84 ± 0.2	1.01 ± 0.2	0.91 ± 0.2	1.10 ± 0.5

Values are presented as mean ± SD; *MAP* mean aortic pressure

**Fig. 4:**
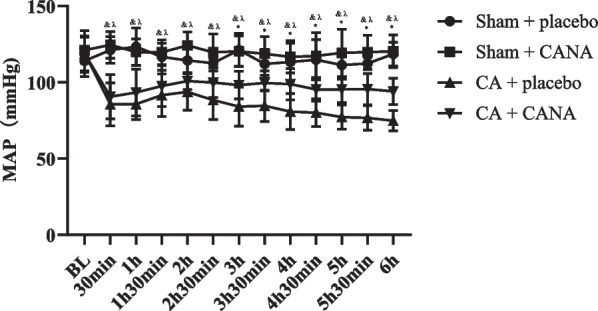
Mean aortic pressure changes in mean arterial pressure during post-resuscitation in 4 groups (each group contains 6 rats); &*P* < 0.05, Sham + placebo vs. CPR + placebo; λ*P* < 0.05, Sham + placebo vs. CPR + CANA; *P < 0.05, CPR + placebo vs. CPR + CANA

To investigate the cardioprotective effect of CANA, we established a CA/CPR rat model and CANA or an equivalent volume of placebo was intravenously administered 15 min after ROSC. Invasive arterial blood pressure is the most commonly used and basic index of hemodynamic measurement, and mean arterial pressure (MAP) was recorded every 30 min. We found that CANA can improve hemodynamics after resuscitation. All rats that received CPR had a significant decrease in blood pressure, which lasted for a period of time and gradually recovered at about 1 h after resuscitation. However, MAP in the control group showed a downward trend in the late stage of resuscitation, but CANA could prevent this trend and keep the blood pressure stable in the late stage of resuscitation. The difference in blood pressure between CPR + placebo and CPR + CANA groups was statistically significant (*P* < 0.05) at 3 h (Fig. 4).

**Fig. 5:**
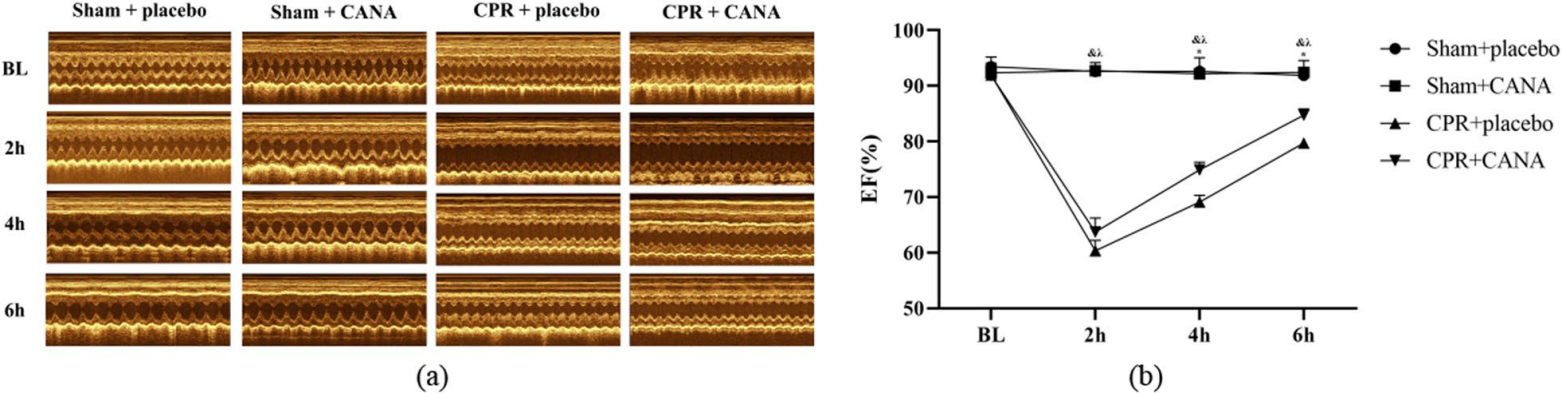
Echocardiography. **a** Echocardiograms of rats in each group; **b** ejection fraction (EF); &*P* < 0.05, Sham + placebo vs. CPR + placebo; λ*P* < 0.05, Sham + placebo vs. CPR + CANA; *P < 0.05, CPR + placebo vs. CPR + CANA

Figure 5 shows myocardial function in rats. Following resuscitation, model groups exhibited significant impairment in left ventricular ejection fraction (LVEF) at 2 h compared to baseline. As time progressed, cardiac function gradually improved in both groups that underwent CA/CPR procedures. Notably, animals treated with CANA demonstrated significantly less impaired cardiac function at 6 h after ROSC compared to the control group (*P* < 0.05).

**Fig. 6:**
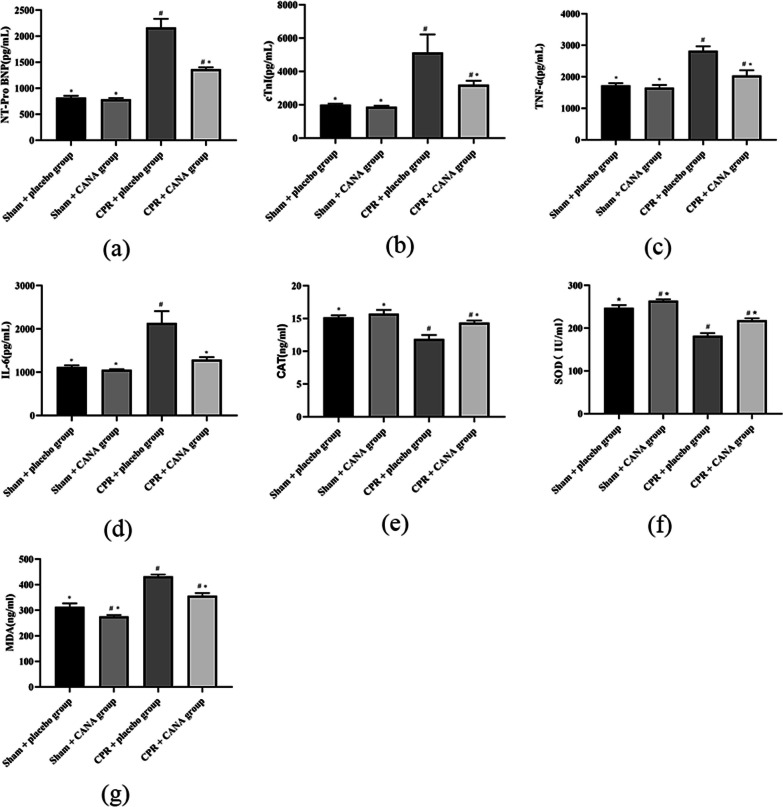
Myocardial injury, inflammation and oxidative stress index. **a** NT-Pro BNP expression at 6 h after return of spontaneous circulation in four groups; **b** cTnI expression at 6 h after return of spontaneous circulation in four groups; **c** TNF-α expression at 6 h after return of spontaneous circulation in four groups; **d** IL-6 expression at 6 h after return of spontaneous circulation in four groups; **e** CAT expression at 6 h after return of spontaneous circulation in four groups; **f** SOD expression at 6 h after return of spontaneous circulation in four groups; **g** MDA expression at 6 h after return of spontaneous circulation in four groups; #*P* < 0.05 vs. the Sham + placebo group. *P < 0.05 vs. the CPR + placebo group

**Fig. 7:**
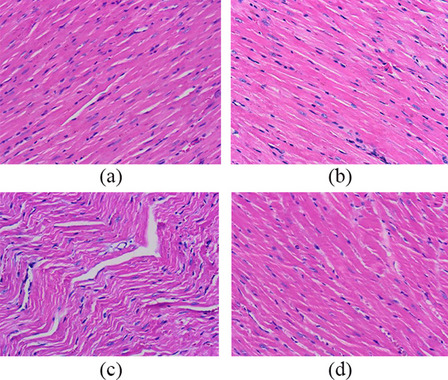
Myocardial pathological slices. Normal myocardial tissue morphological in Sham + placebo group (**a**) and Sham + CANA group (**b**), representative myocardial tissue morphological injuries in the CPR + placebo (**c**) and CPR + CANA group (**d**). Myocardial injury in the CPR + CANA group was reduced compared with the CPR + placebo group, as characterized by myocardial cell edema, transverse striations disappeared and cell arrangement disorder.

The concentrations of cTnI and NT-proBNP in each group were determined by the ELISA method. We found that the concentrations of cTnI and NT-proBNP were significantly increased in each model group after resuscitation, which was consistent with our previous experimental results. However, the concentrations of cTnI and NT-proBNP in the CPR + CANA group were significantly lower than those in the CPR + placebo group (*P* < 0.05) (Fig. 6). In addition, we further evaluated the myocardial histopathological changes after 6 h of resuscitation by HE staining. We can see that the rats in the sham-operated group had an interconnected network of myocardial fibers, neatly arranged, evenly stained nuclei, and visible striated; In the model group, the intercellular space of myocardial cells increased, the nuclear staining became light or even disappeared, the arrangement of myocardial fibers was disordered, and the transverse striations disappeared. In the CPR + CANA group, the myocardial cell texture was still clear, and the degree of interstitial swelling and cell destruction was reduced (Fig. 7). All these suggest that CANA can ameliorate the myocardial injury in CA/CPR.

Using ELISA to detect indicators of myocardial oxidative stress injury, we found that the levels of SOD and CAT in the myocardial tissue of rats in the sham operation + CANA group were the highest. In the CPR + placebo group, MDA increased significantly (*p* < 0.05), while CAT and SOD decreased significantly (*p* < 0.05). We found that canagliflozin can alleviate this change and reduce the oxidative stress injury in the model group, which was manifested by a significant increase in myocardial tissue SOD and CAT content and a reduction in myocardial tissue MDA content (*P* < 0.05) (Fig. 6).

To determine the level of myocardial inflammation in rats after CPR, we detected the levels of TNF-α and IL-6 in the myocardial homogenate of rats in each group 6 h after CPR by ELISA. The results showed that the contents of TNF-α and IL-6 in the myocardium of the Sham + CANA group were the lowest. However, there was no significant difference between them and the Sham + placebo group. The model group up-regulated the level of myocardial inflammation, and the expression of TNF-α and IL-6 was significantly higher than that of the sham-operated group (*P* < 0.05). CANA significantly decreased the levels of TNF-α and IL-6 after CA/CPR (*P* < 0.05), suggesting that CANA can reduce the inflammatory response after cardiopulmonary resuscitation (Fig. 6).

**Fig. 8:**
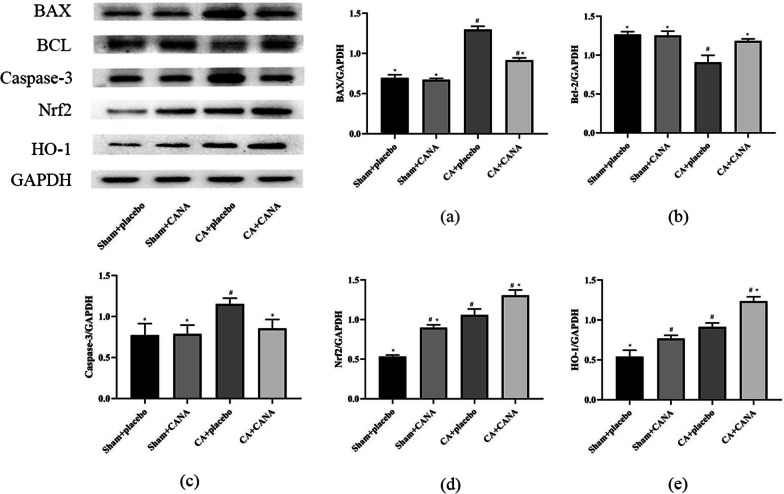
Apoptosis index and Nrf2/HO-1 signaling pathway. **a** BAX expression at 6 h after return of spontaneous circulation in 4 groups; **b** Bcl-2 expression at 6 h after return of spontaneous circulation in four groups; **c** Caspase-3 expression at 6 h after return of spontaneous circulation in four groups; **d** Nrf2 expression at 6 h after return of spontaneous circulation in four groups; **e** HO-1 expression at 6 h after return of spontaneous circulation in four groups; #*P* < 0.05 vs. the Sham + placebo group. *P < 0.05 vs. the CPR + placebo group

To determine whether cardiac dysfunction after cardiopulmonary resuscitation is related to apoptosis, we detected the expression of apoptotic protein and anti-apoptotic protein in myocardial tissue homogenate of rats in each group by Western blotting 6 h after resuscitation. We found that the level of cardiomyocyte apoptosis in the CPR + placebo group was significantly higher than that in the Sham + placebo group, suggesting that apoptosis is involved in cardiac dysfunction after resuscitation. CANA could reduce CPR-induced apoptosis, and the expression of BAX and caspase-3 was significantly decreased, and the expression of Bcl-2 was significantly increased compared with the placebo group (*P* < 0.05) (Fig. 8).

More and more studies have shown that the Nrf2/HO-1 signaling pathway is important in oxidative stress and also closely related to inflammation and apoptosis. Therefore, we measured protein levels of key signaling pathway molecules, namely, Nrf2 and HO-1. We found that both CANA and I/R injury could up-regulate the expression of Nrf2 and HO-1 protein. However, the endogenous antioxidant effect regulated by CANA was not enough to resist the reperfusion-induced oxidative stress. The CPR + CANA group significantly increased the content of Nrf2 and HO-1 (*P* < 0.05), which increased this advantage, reduced oxidative stress injury in the model group, and even potentially regulated the inflammatory response and apoptosis. However, the specific regulatory mechanism needs to be further verified by cell experiments (Fig. 8).

## Discussion

This study investigated whether intravenous injection of CANA during the initial resuscitation period can improve cardiac function after cardiac arrest in a CA/CPR rat model. The present study demonstrated that the administration of CANA significantly ameliorated PRMD induced by cardiac arrest and CPR with decreased myocardial injury score, increased MAP, decreased levels of cTnI and NT-ProBNP. We also found that CANA directly suppressed the expression of inflammatory cytokines (IL-6, TNF-α), reduced oxidative stress and apoptosis via a Nrf2/HO-1-dependent mechanism. In the rat model of cardiac arrest and CPR, CANA attenuated oxidative stress and inhibited pro-inflammatory and pro-apoptotic signaling pathways, providing a potentially novel mechanism underlying the protective effects against PRMD.

Cardiac arrest is a common clinical emergency. The heart stops beating and the blood supply is cut off, leading to acute organ failure throughout the whole body. Patients who do not receive effective medical care often die within this short period of time. CPR is a life-saving intervention that successfully restores circulation and cardiac function in patients with sudden cardiac arrest (SCA). Unfortunately, the outcomes of CPR are disappointing. Post-cardiac arrest syndrome (PCAS) is an independent risk factor that affects the survival rate of resuscitated patients and is closely related to prognosis [27]. Myocardial dysfunction is the most common manifestation of PCAS and the leading cause of early death in patients with cardiac arrest. More than 68% of patients develop PRMD [4, 6]. Therefore, it's of great value to identify novel therapeutic strategies to improve PRMD after CA/CPR.

In clinical studies, several sodium–glucose cotransporter 2 inhibitors (SGLT2i) have shown different cardiovascular advantages in individuals with heart failure, myocardial infarction, arrhythmia, and cardiomyopathy [28–30]. In addition, this cardioprotective effect was independent of glucose management. In other words, SGLT2 inhibitors showed cardioprotective benefits in non-diabetic subjects. The use of CANA in heart failure patients regardless of pre-existing diabetes is recommended in the CHIEF-HF clinical trial [31]. In recent EMMY study, empagliflozin significantly enhanced cardiac outcomes in patients with acute myocardial infarction following 72-h intervention [32]. In contrast to local *I*/*R* in myocardial infarction, in this study we investigated the cardioprotective effect of acute intravenous injection of CANA on systemic *I*/*R* injury after CA/CPR.

Currently, the most commonly used animal models of CA/CPR include asphyxia, electric shock ventricular fibrillation and the high potassium method [33]. Relevant studies have shown that rapid ventricular arrhythmias, including ventricular fibrillation and ventricular tachycardia, are the most common in sudden cardiac death (SCD) with complete ECG recordings [34]. Therefore, electric shock ventricular fibrillation seems more aligned with clinical practice than other methods. However, the traditional ventricular endocardium electric shock ventricular fibrillation method requires highly professional operation and has relatively significant trauma. In the present study, we used an improved trans epicardial electrical stimulation to establish a standard rat model of VF-induced cardiac arrest and resuscitation. In addition, we determined the dose of intravenous CANA based on available studies (3 ug/kg) [35]. Our study was the first study to investigate the effects of CANA on myocardial dysfunction after VF-induced cardiac arrest and CPR model in rats.

PRMD is a common complication in post-resuscitation patients, manifesting mainly as systolic and diastolic dysfunction, arrhythmias and recurrent cardiac arrest. This cardiac dysfunction is usually reversible and recovery is possible within 48–72 h. Approximately 2/3 of patients with VF-induced cardiac arrest develop circulatory dysfunction during the course of the disease, requiring pharmacological or mechanical intervention. However, failure to treat these disturbances promptly will result in an adverse outcome after cardiac arrest [36]. Myocardial tissue mild I/R injury can release cTnI and NT-ProBNP, which are routinely used in the clinic as indicators of early myocardial damage. Moreover, the concentrations of cTnI and NT-ProBNP indicate the severity of the myocardial injury [37, 38]. In our study, there was a significant increase in both cTnI and NT-ProBNP concentrations in the CA/CPR model group 6 h after resuscitation. The canagliflozin therapy intervention significantly reduced this rising trend, myocardial injury biomarkers in CPR + CANA group higher than sham surgery controls while significantly lower than CPR + placebo group. On echocardiography, LVEF showed similar changes. The left ventricular function decreased significantly after CA/CPR and improved gradually with time. CANA can accelerate the recovery of left ventricular function. The change suggests that CANA can ameliorate myocardial I/R injury. The same change was also confirmed in myocardial pathological sections. In the sham surgery group, the morphology and structure of myocardial cells were normal, and no inflammatory cell infiltration was observed. However, in the CPR + placebo group, edema of myocardial interstitial substance, light staining or even disappearance of the nucleus, and disorder of myocardial fibre arrangement were observed. Compared with the CPR + placebo group, CANA treatment significantly reduced the myocardial fibre swelling and distortion in CA/CPR rats.

Previous studies have shown that ROS can be generated immediately after ischaemia, and various ROS affect normal physiological functions of cells by damaging protein, lipid and even DNA structure [39, 40]. In addition, oxidative stress is a critical factor in the occurrence and development of cardiac I/R injury. Oxidative stress also links pro-inflammatory response and apoptosis to aggravate myocardial cell injury, except for direct toxic injury [41, 42]. In addition to glucose-lowering effects, antioxidant properties of SGLT inhibitors have been demonstrated [23, 43, 44]. As a highly selective inhibitor of SGLT-2, CANA reduces endothelial injury to slow coronary atherosclerosis by reducing ROS production and blocking oxidative stress signaling pathway (EGFR/Src/Rho-kinase) mediated by ROS [22]. Based on previous studies, we further verified that CANA can protect I/R cardiac by reducing oxidative stress. In this study, we found that the expression of oxidative markers was significantly increased in CA/CPR rat myocardium compared with the sham-operated group after 6 h of resuscitation. However, its expression in CANA + CA/CPR group showed a significant downward trend. We found that the expression of antioxidant enzymes in the myocardium of I/R rats was significantly upregulated after treatment with CANA.

During cardiac arrest, ischaemia, hypoxia and disruption of the energy chain drive injured cardiomyocytes to release a variety of DAMPs. After hearts pumping recovery, innate immune cells recognise DAMP and activate the TLR pathway to release excessive inflammatory factors and chemokines, causing inflammatory damage [45]. TNF-α and IL-6 are master cytokines of the inflammatory response. Under pathological conditions, myocardial cells and macrophages secrete TNF-α, which combines with membrane TNFR1 to form a complex. This complex participates in inflammation-mediated cell injury process, and also participates in pathophysiological reactions, such as the induction of cardiomyocyte apoptosis and the regulation of ventricular remodelling [46]. In addition, TNF-α can activate NF-KB, which further activates TNF-α, IL-6, and other inflammatory factors to form an inflammatory cascade response. IL-6 is a multi-effect secondary cytokine, often playing an anti-inflammatory role in the acute phase and gradually becoming pro-inflammatory after prolonged activation. It can mediate the release of oxygen-free radicals and proteolytic enzymes, regulate the excitation–contraction coupling of cardiomyocytes and promote myocardial fibrosis to damage the normal myocardial function [47]. Multiple meta-analyses have shown that IL-6 is significantly associated with all-cause mortality in multiple cardiovascular diseases [48, 49]. In this study, we found that the expression of relevant inflammatory factors (TNF-α, IL-6) in cardiac myocytes of CA/CPR rats was significantly increased after 6 h of resuscitation, consistent with previous studies [50]. In addition, CANA can significantly alleviate the expression of the above inflammatory factors. Previous studies have reported the anti-inflammatory performance of CANA in chronic kidney disease, fatty liver, atherosclerosis, myocarditis, and other diseases [51–54], showing the independent anti-inflammatory properties of CANA. Our results have shown that attenuation of the inflammatory response is also one of the mechanisms by which CANA alleviated myocardial injury after resuscitation.

Arrhythmias, myocardial stunning, no-reflow phenomena, and myocardial cell death due to reperfusion are the most common manifestations of myocardial *I*/*R* injury [55], whereas apoptosis is the most common form of reperfusion injury-induced cardiomyocyte death. Some studies have shown that apoptosis is not associated with myocardial dysfunction after resuscitation [56]. However, recent research has shown that prostaglandin E1 (PGE1) inhibits caspase-mediated apoptosis following resuscitation by reducing the opening of mitochondrial permeability transition pores (mPTP) in CA/CPR rat model and the H9C2 cells hypoxia/reoxygenation model [57]. Similarly, in an experimental study on porcine, caspase-3 expression and the number of TUNEL-stained positive cells in cardiac tissue 24 h after resuscitation were considerably greater in the CA/CPR group than in the sham group [58]. Apoptosis is a process in which cells die to adapt to their surroundings. It involves many signaling pathways, including the Fas/FasL pathway-mediated exogenous pathway and the mitochondria-mediated endogenous pathway, and caspase-3 is the final common pathway in most cases. In our investigation, we discovered that the expression of anti-apoptotic proteins BAX and caspase-3 was significantly decreased in the CPR + placebo group. In contrast, the expression of the apoptotic protein Bcl-2 was increased. However, canagliflozin could alleviate this apoptosis trend in I/R injury. We found that the degree of myocardial apoptosis in the CPR + CANA group was significantly decreased.

SGLT2 inhibitors have shown good cardiovascular benefits in many clinical trials. In diabetic patients, they can significantly improve early hemodynamics, direct myocardial protection (regulate myocardial energy metabolism, anti-myocardial fibrosis, etc.) and vascular protection (improve vascular endothelial function, regulate vascular remodeling, etc.) to improve cardiovascular event outcomes [23, 59–62]. As the cardiovascular protective effects independent of glucose-lowering effects are increasingly recognized, researchers redirect the focus of studies to non-diabetic patients, particularly in ischemic heart disease. Sabe et al. found that in a swine model of chronic myocardial ischaemia, CANA can reduce ischemic area and improve myocardial function through JAK/STAT pathway, AMPK pathway, and oxidative signaling pathways [63]. In a model of regional myocardial *I*/*R*, researchers also found that CANA can improve ischemic myocardium contractile function [64, 65]. However, most animal studies used CANA pretreatment with an intragastric administration time as long as several weeks, which was inconsistent with the actual situation in clinical emergency patients. Moreover, most of the experiments used the coronary artery ligation method, which could only induce local myocardial ischaemia but could not cause global *I*/*R* injury. Research about whether the acute administration of CANA alleviates myocardial dysfunction after whole-heart *I*/*R* is still at an early stage, and the underlying molecular mechanisms are unknown. The nuclear factor-erythroid 2-related factor 2 (Nrf2) signaling pathway plays an important role in *I*/*R* injury. Nrf2 and Keap1 form a complex in the cytoplasm under physiological circumstances. When oxidative stress products such as ROS are stimulated, Nrf2 dissociates from Keap1, increases nuclear translocation of Nrf2, binds to nuclear antioxidant response element AER, and induces downstream antioxidant stress gene transcription (such as HO-1), which initiates endogenous antioxidant pathways and maintains intracellular redox balance. Both I/R injury and CANA were observed to promote the expression of Nrf2 in our investigation. Endogenous antioxidant mechanisms, however, are unable to counteract the huge quantity of ROS produced during reperfusion. We discovered that intravenous use of CANA in the resuscitated rat significantly increased Nrf2 expression, activated the Nrf2/HO-1 signaling pathway, and ameliorated oxidative stress damage. Hasan et al. 's et al. also found that canagliflozin can reduce cardiac oxidative stress injury through the Nrf2/HO-1 pathway in the isoproterenol (ISO)-induced cardiac injury model [66]. Neutrophil infiltration and inflammatory cytokine release are critical steps in the development of IRI. Activation of the Nrf2/HO-1 pathway can alleviate *I*/*R* injury and inflammatory responses in tissues or organs. Some investigations have revealed that Nrf2 is a significant mediator linking the inflammatory response and oxidative stress, although its precise molecular mechanism remains unknown. Currently, relevant research focuses mostly on the regulation of Nrf2 and NLRP3 inflammasomes [67, 68]. For example, Hou et al. found that activation of the NLRP3 inflammatory complex plays an important role in inflammatory damage in brain *I*/*R* injury, and Nrf2 inhibits activation of the NLRP3 inflammatory complex by regulating the Trx1/TXNIP complex, thus playing a role in alleviating *I*/*R* injury [69]. The anti-inflammatory effect of the Nrf2 pathway may also be related to the NF-κB and MAPK pathways. Nrf2 has been shown to reduce the production of inflammatory factors and the occurrence of inflammatory responses by inhibiting the activation of NF-κB and MAPK. Furthermore, the downstream protein HO-1 promotes macrophage change from pro-inflammatory to anti-inflammatory [70, 71]. Its catalytic products have anti-inflammatory properties. Yan et al. discovered a more severe inflammatory response in both the I/R model of Nrf2^−/−^mice and the hypoxia/reoxygenation cell model with Nrf2 gene silencing in research on *I*/*R*-induced lung injury [72]. Reduced oxidative stress and inflammatory response can minimize cardiac cell damage and apoptosis, and the researchers have also found that activation of the Nrf2/HO-1 signaling pathway can reduce apoptosis following myocardial I/R[73, 74]. Lu et al. reported that Artesunate reduces apoptosis by up-regulating the expression of the anti-apoptotic transcription factor Nrf2 and down-regulating the ROS-dependent p38MAPK pathway [75]. Wang et al. found that neohesperidin can inhibit apoptosis and oxidative stress through activation of the Akt/Nrf2/HO-1 pathway, which can have a neuroprotective effect on cerebral IRI with neuroprotective effects [76]. Induced HO-1 expression in retransplanted hearts could significantly reduce the number of apoptotic cells. In addition, inhibition of HO-1 activity reversed this protective effect [77, 78]. Keap1/Nrf2/HO-1, SIRT1/Nrf2/HO-1 and others are important pathways to attenuate *I*/*R* injury [79–81]. As a result, we postulated that the anti-inflammatory, anti-oxidative stress and anti-apoptotic effects of CANA in myocardial *I*/*R* may be directly or indirectly regulated by the Nrf2/HO-1 signaling pathway.

Our study investigated the effects of acute administration of CANA after cardiac arrest on ischemic myocardium. We found that canagliflozin has a cardioprotective effect independent of its glucose-lowering effect, can improve the cardiac function of rats after resuscitation, and reduce the myocardial pathological changes induced by *I*/*R*. The specific mechanism may be related to the regulation of the Nrf2/HO-1 signaling pathway to improve myocardial antioxidant and anti-inflammatory capacity, and then reduce apoptosis. These results indicate that CANA is a potential drug the prevention and treatment of *I*/*R* injury and provide an experimental basis for the clinical application of CANA in ischemic cardiomyopathy.

### Limitations

This study has several limitations. First, the experimental animals had no underlying disease, which is not consistent with the high-risk populations for cardiac arrest; second, CANA was administered at ROSC 15 min, which is not consistent with actual clinical administration; third, the CA + CANA + Nrf2 inhibitor group was not set up to verify the role of the Nrf2/HO-1 pathway; fourth, no corresponding cell experiments were performed; finally, the sample size of this study was relatively small.

## Conclusions

We demonstrated for the first time that the acute use of canagliflozin alleviated post-resuscitation myocardial dysfunction after cardiac arrest and CPR in the rat model. The underlying mechanism could be through Nrf2 signaling pathway while suppressing proinflammatory and proapoptotic pathways. Our work describes canagliflozin, elucidates the potential mechanism of its cardioprotective effect, and in the future, it may be used to reduce post-resuscitation myocardial dysfunction in patients resuscitated by cardiac arrest and CPR.

## Data Availability

The data sets used and analysed during the current study are available from the corresponding author on reasonable request.

## Abbreviations

CA: Cardiac arrest
CANA: Canagliflozin
ROSC: Restore of spontaneous circulation
ECG: Electrocardiogram
MAP: Mean arterial pressure
LVEF: Left ventricular ejection fraction
I/R: Ischaemia/reperfusion
CPR: Cardiopulmonary resuscitation
PRMD: Post-resuscitation myocardial dysfunction
DAMPs: Danger-associated molecular patterns
ROS: Reactive oxygen species
T2DM: Type 2 diabetes mellitus
VF: Ventricular fibrillation
CO2: Carbon dioxide
SCA: Sudden cardiac arrest
PCAS: Post-cardiac arrest syndrome
SGLT2i: Sodium–glucose cotransporter 2 inhibitors
SCD: Sudden cardiac death
PGE1: Prostaglandin E1
